# Phytochemical investigations and antiproliferative secondary metabolites from *Thymus alternans* growing in Slovakia

**DOI:** 10.1080/13880209.2017.1291689

**Published:** 2017-02-21

**Authors:** Stefano Dall’Acqua, Gregorio Peron, Sara Ferrari, Valentina Gandin, Massimo Bramucci, Luana Quassinti, Pavol Mártonfi, Filippo Maggi

**Affiliations:** a Department of Pharmaceutical and Pharmacological Sciences, University of Padova, Padova, Italy;; b School of Pharmacy, University of Camerino, Camerino, Italy;; c Department of Botany, Institute of Biology and Ecology, P. J. Šafárik University, Košice, Slovakia

**Keywords:** Triterpene, flavonoids, essential oil, GC-MS, HPLC-MS

## Abstract

**Context:***Thymus alternans* Klokov (Lamiaceae) is a neglected species of the genus *Thymus* (Sect. *Serpyllum*) endemic to Carpathian area, where it is used as a flavouring agent and for medicinal purposes.

**Objective:** The aim of the work was to identify antiproliferative constituents from the flowering aerial parts of this plant.

**Materials and methods:***Thymus alternans* extracts were analyzed by HPLC-MS^n^ and subjected to extensive chromatographic separations. The isolated compounds (phenolics and triterpenes) were structurally elucidated by MS and 1D and 2D NMR experiments. Essential oil (EO) composition was determined by GC-FID and GC-MS. Six purified triterpenes and EO were assayed for *in vitro* antiproliferative activity against a panel of human cancer cells, namely, breast (MDA-MB 231), colon (HCT-15 and HCT116), lung (U1810), pancreatic (BxPC3), melanoma (A375) and cervical carcinoma (A431) cells.

**Results:** The structures of the isolated compounds were achieved on the basis of H-NMR and MS experiments. Luteolin-4′-*O*-β-d-glucopyranoside (P1), chrysoeriol-7-*O*-β-d-glucopyranoside (P2), chrysoeriol-5-*O*-β-d-glucopyranoside (P3), apigenin-7-*O*-β-d-glucopyranoside (P4), rosmarinic acid (P5), rosmarinic acid-3′-*O*-β-d-glucopyranoside (P6), caffeic acid-3-*O*-β-d-glucopyranoside (P7), 3*α*-hydroxy-urs-12,15-diene (T1), α-amyrin (T2), β-amyrin (T3), isoursenol (T4), epitaraxerol (T5), and oleanolic acid (T6). GC-MS analysis revealed that the EO of *T. alternans* was devoid of phenols and belonged to the nerolidol-chemotype, that is typical of the Sect. *Serpyllum*. The six purified triterpenes (T1-T6) were active with IC_50_ ranging from 0.5 to 5 μM being comparable or better than those of reference compounds betulinic acid and cisplatin. The EO exhibited significant effects on A375, MDA-MB 231 and HCT116 cell lines with IC_50_ in the range of 5–8 μg/mL.

**Conclusion:** The reported results suggest that *T. alternans* can be considered as a good source of phytoconstituents with possible importance in the pharmaceutical field.

## Introduction

The genus *Thymus* L. (Lamiaceae) includes 215 species divided in 8 sections, with the evolutionary centres in the Mediterranean area from where it spreads to Europe, Asia, Northern America and Abyssinia, as well as to Greenland (Morales 2002). Many species of this genus are used throughout the world in the traditional medicine and are nowadays important species in food, cosmetics and pharmaceutics (Nabavi et al. 2015). For this reason, the evaluation of the phytochemical composition of different *Thymus* species can be considered fundamental for possible uses of the different plants in pharmaceutical, cosmetic and food products. A large number of studies were conducted especially on *T. vulgaris* L., a spice used in many regions of the world and one of the most important herbal drugs used as an antibacterial agent (Nabavi et al. 2015). However, several species of the genus remain unexplored and deserve attention of scientists. Among them *T. alternans* Klokov is endemic to Carpathian regions and can be considered a neglected species of the genus *Thymus* (Klokov 1960). *Thymus alternans* grows in dry meadows and hilly areas of Carpathian regions up to 1100 m above the sea level (Mártonfi 1996; Načhyčhko 2014). This species belongs to the Sect. *Serpyllum* (Miller) Bentham, Subsect. *Alternantes* Klokov, and taxonomically is widely accepted by specialists of the genus *Thymus*, as well as in the IPNI (The International Plant Names Index, www.ipni.org) database. Actually, *T. alternans* is recognized as a well distinguished taxon, characterized by sympodial branching with terminal fertile branch and alelotrichous indument (Mártonfi 1996). The taxon belongs to the group of tetraploid taxa with 2 *n* = 4 × = 56 (Mártonfi & Mártonfiová 1996) and is probably closely related to the species *T. nummularius* M. Bieb. and *T. pseudonummularius* Klokov et Des.-Shost. from Caucasus.

*Thymus alternans* has been frequently employed in the folk medicine of the regions in which occurs, often together with *T. pulegioides* L., with which it shares the same habitat. Owing to the polymorphism occurring in many *Thymus* species, including *T. serpyllum* s.l., which makes their identification very problematic, spontaneous plants of *T. alternans* are often used together with those of *T. pulegioides* (both belonging to the sect. *Serpyllum*) as an ingredient of the drug called ‘*Serpylli herba*’, which is included in the European Pharmacopoeia (2008).

Continuing our research on secondary metabolites from unexplored species of the genus *Thymus* growing in Slovakia (Maggi et al. 2014), the present work reports a comprehensive phytochemical analysis on polar phenolics, triterpenes, and volatile constituents obtained from the flowering aerial parts of *T. alternans* growing in Slovakia. Extracts obtained with solvents of increasing polarity were analyzed by HPLC-MS^n^ and were further subjected to extensive chromatographic separations, leading to the isolation of 12 compounds that were fully characterized by 1D and 2D NMR. Furthermore, hydrodistilled essential oils (EOs) from different samples were obtained and the quali-quantitative compositions were carefully investigated by GC-FID and GC-MS. Finally, the purified triterpene constituents as well as the EO were assayed for *in vitro* cytotoxic activity on a panel of human cancer cell lines.

## Materials and methods

### Plant material

**Figure 1.**
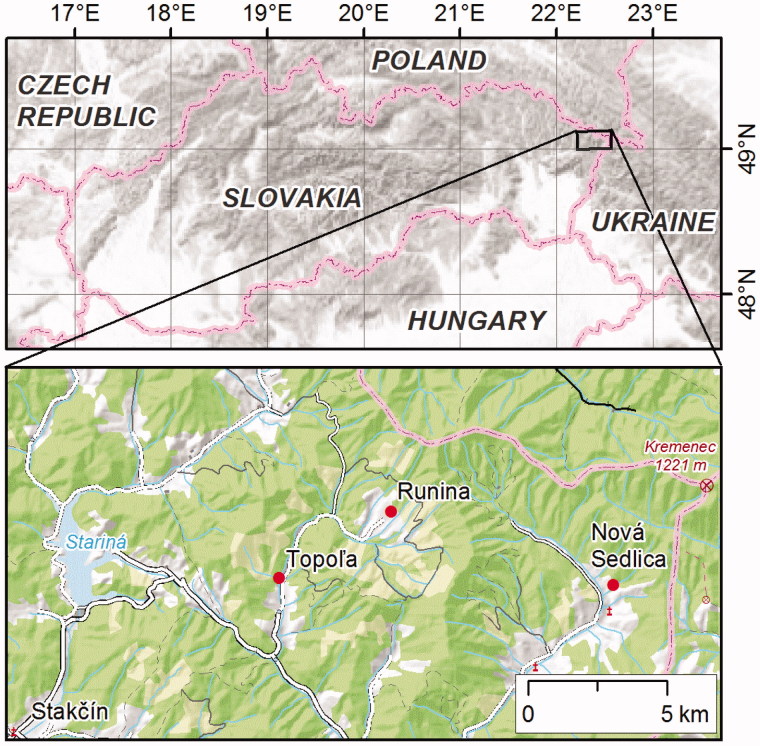
Distribution of the collection sites of *T. alternans* in Slovakia.

Aerial parts of *T. alternans* were collected in the time of flowering on 16 June 2014 by Albert Rákai and Erik Ducár in three different localities in Bukovské vrchy hills in eastern Slovakia (Figure 1). These localities are: 1. the village of Runina, path across the fields; 49°4’32.34” N; 22°24’29.68” E; 572 m a.s.l. (voucher specimen KO 31164); 2. the village of Topoľa, near the bridge over the small river ‘Ulička’; 49°3’11.46” N; 22°21’14.32” E; 398 m a.s.l. (KO 31165); 3. the village of Nová Sedlica, path across the forest; 49°3’14.95” N; 22°31’8.13” E; 452 m a.s.l. (KO 31166). Plants were identified by the specialist of the genus *Thymus*, Pavol Mártonfi, and voucher specimens are deposited in the KO herbarium (Herbarium of the Botanical Garden, P. J. Šafárik University, Košice, Slovakia). Plant samples were then stored in absence of light and at r.t. (ca. 22 °C) until completely dry.

### HPLC, HPLC-MS, flash chromatography and semi-preparative HPLC

Silica gel plates (cod. 5171 Merck) and silica gel (60 mesh) were acquired from Sigma (Milan, Italy). Solvents were acquired from Carlo Erba (Milan). Varian Intelliflash flash chromatograph was used for preparative chromatography. A Varian 920 liquid chromatograph was used for semi-preparative HPLC. ESI-MS measurements were performed on a Varian 500 MS ion trap spectrometer. HPLC-DAD analyses were performed on an Agilent 1100 chromatographic system with Diode Array (1100 series). HPLC-MS was performed using a Varian 212 binary chromatograph hyphenated with a Varian 500 MS ion trap spectrometer.

### NMR analysis

1D and 2D NMR spectra were obtained on Bruker AVANCE 300 MHz and Bruker AVANCE III 400 MHz spectrometers, dissolving the samples in deuterated methanol. 2D experiments, namely, COSY, HSQC-DEPT, HMBC, TOCSY and NOESY were performed on a Bruker AVANCE III 400 MHz spectrometer and were used for structure elucidation.

### Analysis of nonvolatile constituents

Aerial parts of *Thymus alternans* (130 g dry material) collected in Nová Sedlica were chopped and extracted with *n*-hexane (100 mL) at room temperature for 10 min in an ultrasound bath (NE extract). Extraction was repeated twice yielding a brown residue. Solvent was removed and the residue was extracted with dichloromethane (100 mL) at room temperature for 10 min in an ultrasound bath, repeating the extraction twice (DM extract). Residual plant material was then extracted with methanol (100 mL) in an ultrasound bath, repeating the extraction twice (ME extract). Solvents were removed under vacuum, yielding a semi-solid dark-brown residue (NE 0.6%, DM 1.88%, ME 3.4%). Preliminary investigation on NE extract showed the presence of lipids, thus this fraction was discharged. DM extract (2.11 g) was applied to a pre-packed silica-gel column Supel Flash Cartridge, 80 g, 40–60 μm silica. Loaded column was eluted with cyclohexane (2 column volumes), then gradually adding acetone (up to 50%) to increase polarity of the eluent. Finally, the column was eluted with 100% methanol. Ninety-eight fractions (25 mL) were collected and pooled on the basis of their TLC chromatographic behaviour in nine different fractions: A (101.4 mg), B (27.4 mg), C (849.7 mg), D (80.2 mg), E (196 mg), F (94.7 mg), G (345.1 mg), H (397.2 mg), I (212.1 mg). The purification of fractions E, F, G, H by semi-preparative HPLC was performed using a PLRP-S 100 Å column (8 μm) as stationary phase. HPLC conditions were as follows: 0.1% formic acid in water (A) and acetonitrile (B) were used as eluents; gradient elution started from 20% (B), then in 30 min to 80% (B); the flow rate was 1.5 mL/min and the injection volume was 100 μL. Monitored wavelength was 205 nm. Isolated compounds were T1 (22 mg), T2 (15 mg), T3 (12 mg), T4 (10 mg), T5 (10 mg) and T6 (25 mg); NMR assignments are reported in supplementary material.

ME extract (1.9 g) was charged on Supel Flash Cartridge, 120 g, 40–60 μm silica and eluted in isocratic mode with a 10:5:0.5 mixture of dichloromethane, methanol and water, respectively. Fifty fractions were collected (50 mL) and then pooled in 10 fractions on the basis of TLC behaviour. Fractions 4 (450 mg), 5 (300 mg) and 6 (350 mg) were then used for semi-preparative HPLC. Semi-preparative reverse-phase HPLC was performed using an Agilent XDB C-18 (2.1 × 150 mm, 5 μm) stationary phase and eluting the column with a gradient of 1% formic acid in water (A) and acetonitrile (B), as follows: 0 min, 10% B; 30 min, 85% B; 35 min, 85% B. Flow rate was 4 mL/min; the monitored wavelengths were 254, 280 and 350 nm; the injection volume was 100 μL. The isolated compounds were P1 (35 mg), P2 (14 mg), P3 (15 mg), P4 (12 mg), P5 (18 mg), P6 (11 mg) and P7 (14 mg), NMR assignments are reported in Supplementary information.

### Hydrodistillation

Dry aerial parts (187–257 g) were cut into small pieces, inserted into 6 L flasks filled with 3.5 L of deionized water and subjected to hydrodistillation using a Clevenger-type apparatus until no more oil was obtained. The oil yield was determined on a dry weight basis, by calculating the moisture content by leaving plant material in a stove at 105 °C for 8 h. The pure EO was stored in a sealed vial protected from light at −20 °C before chemical analysis and biological assays.

### GC-FID and GC-MS analyses

Volatile components of *T. alternans* were analyzed on an Agilent 4890D instrument coupled to an ionization flame detector (FID). Separation was achieved on an HP-5 capillary column (5% phenylmethylpolysiloxane, 30 m, 0.32 mm i.d.; 0.25 μm film thickness; J and W Scientific, Folsom, CA), using the following temperature program: 5 min at 60 °C, then 4 °C/min up to 220 °C, then 11 °C/min up to 280 °C, held for 15 min. Injector and transfer line temperatures were 280 °C; He was the mobile phase, with a flow rate of 1.8 mL/min; split ratio was 1:34. A standard mixture of aliphatic hydrocarbons (C8–C30; Sigma, Milan) diluted in *n*-hexane was used to determine the retention indices (RIs) of peaks. Oil samples were diluted to 1:100 in *n*-hexane and 1 μL of the solution was injected into GC system. Analyses were performed in triplicate and the mean values were reported. Data analysis was carried out by HP3398A GC Chemstation software (Hewlett Packard, Rev. A.01.01). Quantification of volatiles was performed by peak-area normalization considering the response factors at FID equal to 1. GC-MS analysis was carried out on an Agilent 6890N gas chromatograph equipped with a 5973N mass spectrometer and using a HP-5 MS (5% phenylmethylpolysiloxane, 30 m, 0.25 mm i.d., 0.1 μm film thickness; J & W Scientific, Folsom) capillary column. The temperature program was the same as that reported above. Injector and transfer line temperatures were 280 °C; He was used as the mobile phase, with a flow rate of 1 mL/min; split ratio was 1:50; acquisition mass range was 29–400 *m/z*. Mass spectra were acquired in electron-impact (EI) mode with an ionization voltage of 70 eV. Oil samples were diluted to 1:100 in *n*-hexane, and 2 μL of the solution was injected into GC-MS system. Data analysis was done by MSD ChemStation software (Agilent, Version G1701DA D.01.00). The major EO constituents were identified by comparison with authentic standards available in the laboratory. Otherwise, the peak assignment was carried out by comparison of retention indices (RIs) and mass spectra (MS) with respect to those reported in commercial (Adams 2007; NIST 08 2008; FFNSC 2 2012) and home-made libraries.

### Cell cultures

Human breast (MDA-MB 231), colon (HCT-15 and HCT116), lung (U1810) and pancreatic (BxPC3) carcinoma cell lines together with melanoma (A375) cells were obtained from American Type Culture Collection (ATCC, Rockville, MD). Human cervical carcinoma (A431) cells were kindly provided by Prof. F. Zunino (Division of Experimental Oncology B, Istituto Nazionale dei Tumori, Milan, Italy). Cell lines were maintained in the logarithmic phase at 37 °C in a 5% carbon dioxide atmosphere using the following culture media, containing 10% fetal calf serum (Euroclone, Milan, Italy), antibiotics (100 units/mL penicillin and 100 μg/mL streptomycin) and 2 mM l-glutamine: (i) RPMI-1640 medium (Euroclone) for HCT-15, A431, BxPC3, HCT116 and U1810 cells; (ii) DMEM (Sigma Chemical Co.) for A375 and MDA-MB 231 cells.

### Cytotoxicity assays

The MTT assay (MTT: 3-(4,5-dimethyl-2-thiazolyl)-2,5-diphenyl-2H-tetrazoliumbromide) was used as a relative measure of cell viability. Briefly, (3-8) × 10^3^ cells/well, dependent upon the growth characteristics of the cell line, were seeded in 96-well microplates in growth medium. After 24 h, the medium was removed and replaced with fresh medium containing the compound to be studied at the appropriate concentration (0.1–30 μM for isolated compounds, 1–100 μg/mL for EO). Triplicate cultures were established for each treatment. After 72 h, each well was treated with 10 μL of a 5 mg/mL MTT solution in phosphate-buffered saline (PBS) and, after 4 h of incubation, 100 μL of a sodium dodecylsulfate (SDS) solution in 0.01 M HCl were added. After an overnight incubation, the extent of MTT reduction was quantified spectrophotometrically using a microplate reader by absorbance measurement at 540 nm. The mean absorbance for each drug dose was expressed as a percentage of the control, untreated, well absorbance and plotted vs. drug concentration. Cytotoxicity is expressed as the concentration of compound inhibiting cell growth by 50% (IC_50_). The IC_50_ values, the drug concentrations that decrease the mean absorbance at 570 nm to 50% of that of untreated control wells, were calculated using GraphPad Prism 4 (GraphPad Software, S. Diego, CA). The final value is the mean ± S.D. of at least three independent experiments performed in triplicate.

## Results and discussion

### LC-MS^n^ and NMR analysis

The HPLC-MS^n^ analysis of the methanolic extract from aerial parts of *T. alternans* allowed the identification of several phenolic constituents on the basis of pseudomolecular [M-H]^−^ ions and their relative fragmentation pathways, as summarized in Table 1.

**Table 1.** Non-volatile compounds extracted from *T. alternans*, tentatively identified on the basis of mass fragmentation spectra.

Identified compound	[M-H]^−^ and fragments
Luteolin-3-*O*-glucopyranoside^a^	447, 285,175
Luteolin-7-*O*-glucopyranoside^a^	447, 285,175
Luteolin-7-*O*-rutinoside^a^	593, 285, 175
Chrysoeriol-hexoside	461, 446, 299, 284, 256
Methoxy luteolin-hexoside	461, 447, 285, 256, 175
Chrysoeriol-7-*O*-hexosyl-deoxyhexoside	923, 461, 285, 175
Rosmarinic acid hexoside	521, 367, 359, 191
Esculin^a^	339, 177
Rosmarinic acid^a^	359, 161
Caffeic acid-hexoside	341, 179, 89
3′-*O*-(8″-*Z*-Caffeoyl) rosmarinic acid^b^	537, 493, 359
Caffeic acid derivative	377, 341, 179
	647, 601, 341, 179
3’-*O*-methyl- rosmarinic acid^b^	377, 161, 133, 105
Apigenin-7-*O*-glucopyranoside^a^	431, 269, 175
Rosmarinic acid derivative	493, 359, 161, 133

a Structure identity confirmed by injection of reference compound.

b The compound was assigned on the basis of literature data.

**Figure 2.**
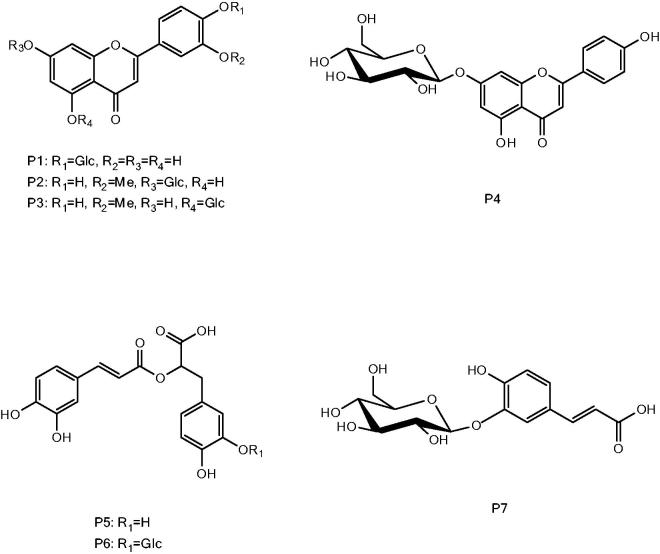
Structures of the isolated phenolic derivatives.

Some of the identified constituents could not be assigned to a single compound due to the presence of possible different isomers and due to the non-availability of reference compounds. Thus, in order to establish correct structures, we decided to isolate them. Chromatographic separations starting from flash chromatography and then using semi-preparative HPLC allowed the isolation of seven phenolic derivatives whose structures were elucidated by 1D and 2D NMR and mass spectrometric analyses (Figure 2). The^1^H, HSQC and HMBC spectra of compound P1 allowed the identification of luteolin, and the presence of a glycosidic residue that was assigned to a glucopyranoside unit. Glycosidation position was deduced from HMBC correlations observed from H-1_glc_ (δ 4.88) and C-4′ (δ 145.2) and from the NOESY correlation observed from H-1_glc_ and H-3′ (δ 6.56); thus the compound P1 was assigned to luteolin-4′-*O*-β-d-glucopyranoside. The compound P2 showed similar NMR data compared to P1 except for the presence of a methoxy signals (δ 3.95) and the glycosidation position that has been revealed by the HMBC correlation observed from the H-1_glc_ and the C-7 (δ 162.3) thus allowing to assign to compound P2 the structure of chrysoeriol-7-*O*-β-d-glucopyranoside (Agrawal & Bansal 1989). Compound P3 was assigned to chrysoeriol-5-*O*-β-d-glucopyranoside (Agrawal & Bansal 1989) on the basis of NOESY correlation observed from H-1_glc_ (δ 6.70) and H-6 (δ 6.96) and due to the HMBC correlation observed from H-1_glc_ and C-5 (δ 160.5). The compound P4 presented the apigenin as aglycone and the glycosidation position was revealed by the NOESY correlations observed from the H-1_glc_ (δ 5.10), H-6 and H-8 (δ 6.82 and 6.51) as well as by the HMBC correlation observed between H-1_glc_ and C-7 (δ 158.6) allowing the assignment to apigenin-7-*O*-β-d-glucopyranoside (Agrawal & Bansal 1989). The rosmarinic acid structure was assigned to P5 due to the presence of a *trans* double bond supported by a pair of doublet at δ 7.51 and 6.26 (*J* = 15.85), as well as to signals ascribable to two 1,3,4 trisubstituted aromatic rings and a 2-hydroxy-propanoic substituent. Compound P6 presented a similar NMR spectrum characterized by signals ascribable to a glucopyranosil moiety that resulted to be linked to the rosmarinic acid to position 3′ due to NOESY correlation observed from the H-1_glc_ (δ 4.30) and H-2′ (δ 6.70) being the rosmarinic acid-3′-*O*-β-d-glucopyranoside P6 (Claire et al. 2005). The structure of caffeic acid-3-*O*-β-d-glucopyranoside was assigned to compound P7 and the glycosidation position was deduced by NOESY correlations from H-1_glc_ (δ 4.37) and H-2 (δ 6.79) (Agrawal & Bansal 1989).

**Figure 3.**
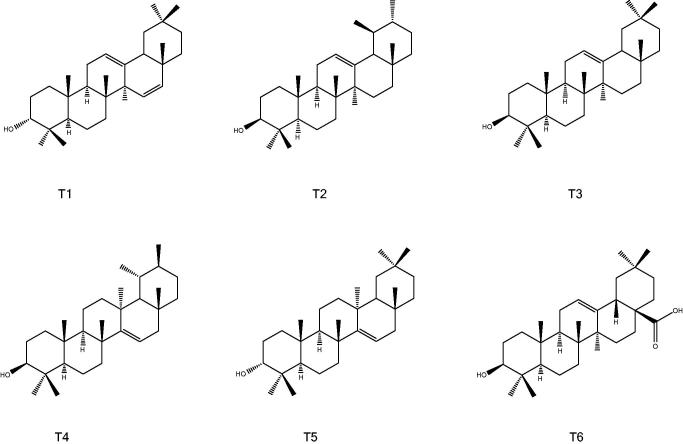
Structures of the isolated triterpenes.

The chromatographic separation from the lipophilic fraction yielded the isolation of six triterpenes (T1-T6) (Figure 3). The NMR spectra (H, HSQC, HMBC, COSY, NOESY) of the different compounds were similar and the analysis and comparison with the literature data allowed the characterization of the isolated compounds. The NMR spectrum of T1 is characterized by the presence of six tertiary methyl groups at *δ* 0.68, 0.69, 0.76, 0.84, 1.01 and 1.25 and two secondary methyl signals 0.79 (3H, d, *J* = 6.5 Hz) and 0.92 (3H, d, *J* = 6.3 Hz,), and a hydroxyl methyne signal at *δ* 3.50 as well as three olefinic protons, two at δ 5.11 and one at *δ* 5.33. ^13^C-NMR shifts and heteronuclear correlations allowed the assignment of structure to 3*α*-hydroxy-urs-12,15-diene, that was previously reported by Gosh et al. (2013) from *Croton bonplandianum* Bail. T2 and T3 were assigned to the well-known α- and β-amyrin (that were also compared with authentic standard available in the lab). ^1^H-NMR spectrum of compound T4 presented six quaternary methyl groups and two secondary methyl groups as doublets at δ 0.93 and 1.02, and a broad doublet at δ 5.50 (assigned to position H-15). The data were in agreement with previously published for isoursenol (Zhang et al. 2008; Shan et al. 2015). Compound T5 was assigned to epitaraxerol characterized by the presence of eight quaternary methyl groups and the olefinic signal at δ 5.50 assigned to position H-15 (Ahiahonu & Goodenowe 2007). Finally, compound T6 was assigned to oleanolic acid by comparison with authentic standard.

### Essential oil analysis

The composition of the EOs hydrodistilled from the flowering aerial parts of *T. alternans* is reported in Table 2. A total of 71 volatile constituents were identified in the three samples analyzed, accounting for 91.0–93.2% of the total compositions. The EOs were mainly composed of terpenoids, notably oxygenated monoterpenes (35.3–39.1%), oxygenated sesquiterpenes (22.5–34.1%), monoterpene hydrocarbons (5.1–15.6%) and sesquiterpene hydrocarbons (12.2–15.1%). The sesquiterpene alcohol (*E*)-nerolidol was the most abundant component in all three oils analyzed (15.8–31.4%). Geranial (6.8–7.7%), linalool (1.7–6.4%), geraniol (3.3–6.2%) and neral (4.9–5.4%) were the most representative compounds among oxygenated monoterpenes. Germacrene D (6.7–7.4%) and (*E*)-β-ocimene (2.6–7.0%) were the predominant compounds among sesquiterpene and monoterpene hydrocarbons, respectively. The three EOs did not show relevant differences in qualitative composition each other. Only quantitative variation was noticed among them. For instance, the population from the village of Topoľa exhibited the highest levels of oxygenated monoterpenes (39.1%) and sesquiterpenes (34.1%), with the major amount of (*E*)-nerolidol (31.4%). On the other hand, the population from Nová Sedlica showed the highest levels of monoterpene hydrocarbons (15.6%) which were mainly represented by (*E*)-β-ocimene (7.0%), and minor amounts of oxygenated sesquiterpenes (22.5%), among which (*E*)-nerolidol attained the lowest level (15.8%).

**Table 2.** Composition of EOs obtained from three populations of *Thymus alternans* growing in Slovakia (NS – village of Nová Sedlica, RUN – village of Runina, POL – village of Topoľa. For details of localities see part Plant material).

N.	Component^a^	RI^b^	RI LIT.^c^	Area%^d^			ID^e^
				NS	RUN	POL	
1	α-Thujene	916	924	0.1	tr^f^		RI, MS
2	α-Pinene	921	932	0.3	0.1	tr	Std
3	Camphene	934	946	0.5	0.1	0.1	Std
4	Sabinene	959	969	tr	tr	tr	RI, MS
5	β-Pinene	962	974	0.3	0.2	0.1	Std
6	1-Octen-3-one	968	972	0.2	0.1	tr	RI, MS
7	1-Octen-3-ol	974	974	1.6	1.1	1.4	Std
8	3-Octanone	979	979	0.2	0.1	0.1	RI, MS
9	Myrcene	981	988	4.0	1.9	1.3	Std
10	3-Octanol	990	988	0.2	0.2	0.1	RI, MS
11	Α-terpinene	1009	1014	0.4	0.3	0.1	RI, MS
12	*p*-Cymene	1017	1020	0.3	0.3	0.1	Std
13	Limonene	1020	1024	0.4	0.3	0.2	Std
14	1,8-Cineole	1021	1026	0.7	0.3	0.4	Std
15	(*Z*)-β-Ocimene	1032	1032	1.0	0.5	0.4	RI, MS
16	(*E*)-β-Ocimene	1041	1044	7.0	4.3	2.6	RI, MS
17	γ-Terpinene	1050	1054	1.0	1.0	0.2	Std
18	*cis*-Sabinene hydrate	1057	1065	2.4	2.3	0.7	RI, MS
19	Terpinolene	1079	1086	0.2	0.2	0.1	Std
20	*trans*-Sabinene hydrate	1089	1098	0.3	0.3	0.1	RI, MS
21	Linalool	1095	1095	2.7	1.7	6.4	Std
22	*n*-Undecane	1100	1100	0.2	0.1		Std
23	1-Octen-3-yl acetate	1110	1110	0.1	0.6	0.1	RI, MS
24	*cis*-*p*-Menth-2-en-1-ol	1113	1118	tr	0.2	0.1	RI,MS
25	*allo*-Ocimene	1124	1128	0.1	tr		Std
26	Camphor	1132	1141	1.1	0.4	1.1	Std
27	*trans*-Chrysanthemal	1141	1153	0.2	0.3	0.2	RI, MS
28	Nerol oxide	1149	1154	tr	0.1	0.1	RI, MS
29	Isoborneol	1154	1155	1.1	0.5	1.0	Std
30	Terpinen-4-ol	1166	1174	1.8	1.8	1.0	Std
31	Isogeranial	1178	1174	0.4	0.4	0.4	RI, MS
32	α-Terpineol	1181	1186	0.5	0.3	0.5	Std
33	Nerol	1222	1227	1.9	4.9	3.1	Std
34	Citronellol	1225	1223	0.1	0.1	0.1	Std
35	Thymol, methyl ether	1229	1232	tr	tr	tr	RI, MS
36	Neral	1234	1235	5.1	5.4	4.9	Std
37	Carvacrol, methyl ether	1236	1241		0.1		RI, MS
38	Geraniol	1251	1249	3.3	6.0	6.2	Std
39	Linalool acetate	1254	1254	2.9	tr	2.4	RI, MS
40	Geranial	1265	1264	6.8	7.7	7.2	Std
41	Isobornyl acetate	1276	1283	tr	tr	tr	Std
42	Carvacrol	1299	1298		0.5		Std
43	δ-Elemene	1325	1335	0.1	0.1		RI, MS
44	Neryl acetate	1360	1359	3.6	1.5	2.3	Std
45	β-Bourbonene	1369	1387	1.8	1.0	1.3	RI, MS
46	Geranyl acetate	1379	1379	0.3	0.9	1.1	RI, MS
47	(*E*)-caryophyllene	1402	1417	1.5	1.0	1.6	Std
48	β-Copaene	1413	1430	0.2	0.2	0.2	RI, MS
49	α-Humulene	1436	1454	0.2	0.4	0.2	Std
50	*allo*-Aromadendrene	1443	1458	0.7	0.1	0.1	RI, MS
51	*cis*-Cadina-1(6),4-diene	1449	1461			0.1	RI, MS
52	(*E*)-β-Farnesene	1450	1454		0.1		RI, MS
53	Germacrene D	1465	1484	6.7	7.4	7.4	RI, MS
54	Bicyclogermacrene	1480	1500	0.5	0.5	0.2	RI, MS
55	α-Muurolene	1487	1500	0.2	tr	0.1	RI, MS
56	β-Bisabolene	1498	1505	1.5	0.7	1.3	RI, MS
57	(*E*,*E*)-α-Farnesene	1501	1505	0.3	0.1	0.1	RI, MS
58	δ-Cadinene	1510	1522	1.1	0.2	0.4	RI, MS
59	β-Sesquiphellandrene	1512	1521	0.2	0.4	tr	RI, MS
60	α-Cadinene	1533	1537	0.1	tr		RI, MS
61	Hedycaryol	1536	1546	2.9	3.6	1.4	RI, MS
62	(*E*)-Nerolidol	1559	1561	15.8	26.4	31.4	Std
63	Germacrene D-4-ol	1559	1574	tr	tr	tr	RI, MS
64	Viridiflorol	1584	1590	0.2			Std
65	γ-Eudesmol	1615	1630	0.4	0.1	0.2	RI, MS
66	*epi*-α-Cadinol	1626	1638	0.4	0.1	0.1	RI, MS
67	*epi*-α-Muurolol	1626	1640	0.4	0.3	0.2	RI, MS
68	β-Eudesmol	1631	1649	0.2	0.2	0.1	RI, MS
69	α-Eudesmol	1635	1652	0.3	0.3	0.2	RI, MS
70	α-Cadinol	1648	1652	1.8	0.8	0.6	RI, MS
71	*n*-Nonacosane	2900	2900	0.1	0.2	0.3	Std
	Total identified (%)			91.0	91.4	93.2	
	Oil yield (%)			0.2	0.2	0.1	
	Grouped compounds (%)						
	Monoterpene hydrocarbons			15.6	9.2	5.1	
	Oxygenated monoterpenes			35.3	34.6	39.1	
	Sesquiterpene hydrocarbons			15.1	12.2	12.9	
	Oxygenated sesquiterpenes			22.5	32.1	34.1	
	Others			2.6	2.4	2.0	

a Compounds are in order of elution from a HP-5MS column.

b Retention index on HP-5MS column.

c Retention index taken from literature (Adams, 2007; NIST 08, 2008; FFNSC 2, 2012).

d Percentage values are means of three determinations, with a RSD% for the main components below 10% in all cases.

e Identification methods: MS, by comparison with the mass libraries Wiley, Adams and NIST 08; RI, by comparison with RIs-containing libraries (Adams, 2007; NIST 08, 2008; FFNSC 2, 2012); Std, by comparison with authentic standard.

f tr, traces (mean value below 0.1%).

The main volatile component of *T. alternans*, the tertiary alcohol (*E*)-nerolidol, is one of the most important volatile components of kiwi (*Actinidia chinensis*), tea (especially Oolong tea), strawberry, grape, snapdragon and maize, and is approved and used as a food flavouring agent by the FDA. Nerolidol is involved in attracting pollinators and in defence against predators (Green et al. 2011). Interestingly, nerolidol is currently under study as a skin penetration enhancer for transdermal drugs. Furthermore, it was shown to inhibit carcinogenesis on azoxymethane-induced neoplasia in rats (Wattenberg 1991), and it was effective as an antioxidant *in vivo* (Nogueira Neto et al. 2013), as an antimicrobial and anti-biofilm agent against *Staphylococcus aureus* (Lee et al. 2014) and *Candida albicans* (Curvelo et al. 2014), and as anti-parasitic versus *Leishmania* species (Arruda et al. 2005) and acari (de Assis Lage et al. 2015). Recently, nerolidol was proven to exert antinociceptive activity mediated by the GABAergic system, and anti-inflammatory activity decreasing TNF-α and IL-1β proinflammatory cytokines (Fonsêca et al. 2016).

The present work constitutes the first investigation on volatile components of *T. alternans.* In all the populations considered in the present work, the phenolic compounds thymol and carvacrol, which are considered as the most frequent EO constituents in *Thymus* taxa, have not been found or have been detected in scarce amount (0.5%). This may be due to adaptation of Slovak populations to low temperatures. Accordingly, it has been reported that the production of phenolic compounds in *Thymus* species is higher in warmer and drier regions, while non-phenolic compounds usually accumulate in higher quantities in colder and damper areas (Keeler & Tu 1991). Therefore, the *T. alternans* populations investigated are to be included in the nerolidol-chemotype, as already reported for other species of the same genus such as *T. serpyllum* (Jamali et al. 2012), *T. caucasicus* (Jamzad et al. 2011), *T. praecox* (Vidic et al. 2010), *T. atticus* (Tzakou & Constantinidis 2005), *T. pulegioides* (Mockute & Bernotiene 2005) and *T. zygioides* (Baser et al. 1999). As regards the related *T. roegneri*, one paper reported on the EO composition of plants growing in Turkey (Tümen et al. 1997). In this case thymol (37.15%), *p*-cymene (12.94%) carvacrol (7.76%) and β-bisabolene (6.96%) were found as the major components, while (*E*)-nerolidol was completely missing. The different chemical profile obtained for *T. alternans* may represent an additional evidence at the basis of the taxonomical differentiation of the two species.

### Cytotoxic activity of isolated triterpenes and EO from thymus alternans on tumour cells

The cytotoxic activity of the six isolated triterpenes and EO isolated from plants collected near the village of Nová Sedlica was evaluated by the MTT assay. The triterpenes (T1-T6) were tested on a panel of five human tumour cell lines including examples of pancreatic (BxPC3), colorectal (HCT-15), cervical (A431), and lung (U1810) cancers as well as of melanoma (A375). For comparison purposes, the cytotoxicity of cisplatin, one of the most widely used anticancer drugs, and of betulinic acid, a naturally occurring triterpene that has been shown to possess a promising anticancer activity (Chudzik et al. 2015), were evaluated under the same experimental conditions. IC_50_ values, calculated from the dose-survival curves obtained after 72 h of drug treatment, are shown in Table 3.

**Table 3.** *In vitro* cytotoxic activity of triterpenes isolated from aerial parts of *T. alternans* collected in Nová Sedlica, Slovakia. Cells (5–8 × 104·mL ^−^ ^1^) were treated for 72 h with increasing concentrations of tested compounds.

IC_50_(μM) ± S.D.					
Compound	A375	HCT-15	A431	BxPC3	U1810
T1	1.57 ± 0.72	0.88 ± 0.22	4.54 ± 1.06	0.52 ± 0.24	2.86 ± 0.83
T2	1.26 ± 0.12	1.46 ± 0.13	4.98 ± 1.14	1.84 ± 0.31	2.25 ± 1.05
T3	0.48 ± 0.67	0.67 ± 0.09	2.03 ± 0.98	0.42 ± 0.13	0.98 ± 0.21
T4	1.45 ± 0.56	1.69 ± 0.72	4.85 ± 1.32	1.42 ± 0.73	3.59 ± 1.31
T5	0.87 ± 0.32	2.42 ± 1.05	3.28 ± 1.09	1.84 ± 0.91	2.93 ± 1.13
T6	0.45 ± 0.10	0.54 ± 0.08	2.16 ± 0.83	0.19 ± 0.09	1.62 ± 0.85
Betulinic acid	ND	6.85 ± 2.18	8.96 ± 2.83	4.28 ± 1.35	ND
Cisplatin	2.06 ± 1.01	15.53 ± 2.48	1.96 ± 0.84	10.17 ± 1.65	0.76 ± 0.85

S.D. = standard deviation. ND = not detected. Cell viability was evaluated by means of MTT test. IC_50_ values were calculated by the dose–response curves by means of four parameter logistic model (*p* < 0.05).

Cytotoxicity data showed that all the considered triterpenes are endowed with a considerable activity, which is greater than that exerted by the reference drug betulinic acid against all tested cancer cell lines. On the other hand, the antiproliferative activity of all isolated triterpenes was even higher than that of cisplatin on melanoma (A375, *p* < 0.05), colon cancer (HCT-15, *p* < 0.05) and pancreatic cancer (BxPC3, *p* < 0.05) cells. Among all isolated derivatives, T3 was the most effective, with a mean IC_50_ value of roughly 0.9 μM, about 6.7 times lower than that calculated for cisplatin (mean IC_50 _=_ _6.1 μM, *p* < 0.05). Similarly, the carboxylated derivative T6 exhibited a cytotoxicity profile comparable to that of T3, with an average IC_50_ value of about 1 μM (*p* < 0.05). Notably, the cytotoxicity of T3 and T6 exceeded that of the metallodrug by a factor of about 25 against HCT-15 colon cancer cells, which are barely chemosensitive to cisplatin. On average, T1, T2, T4 and T5 showed a quite similar pattern of response against all tested cell lines, eliciting average IC_50_ values of about 2 μM, roughly three times lower than those obtained with the reference drugs (mean IC_50_ of 6.7 and 6.1 μM for betulinic acid and cisplatin, respectively, *p* < 0.05).

Considering the similar pair of compounds T2/T3 (differencing each other only in the structure of ring E), T3 showed higher cytotoxic activity against all the tested cell lines compared to T2. Thus, minor changes in the structure of these compounds may induce moderate changes in the observed activity. On the other hand, considering the pair T4/T5, also these differing in the structure of ring E, T5 showed higher activity against A375, A431 and U1810 cell lines compared to T4. Therefore, a systematic Structure Activity Relationship (SAR) study may be useful in order to assess notable key point in the basic structure bearing useful improvement of activity.

Concerning possible mechanism of action on tumour cells, betulinic acid is considered as a promising anticancer agent acting through activation of the mitochondrial pathway of apoptosis in cancer cells (Fulda & Kroemer 2009). On the other hand, no data are available on the mode of action of T1-T6. For this reason, further studies on *T. alternans* triterpenes are needed in order to evaluate their possible role on apoptosis. It is worthy to underline that these compounds are well known for possessing moderate toxic effects when administered orally, thus are considered as significant models for the development of new classes of antiproliferative compounds.

The cytotoxic effects of *T. alternans* EO were evaluated on A375, MDA-MB 231, and HCT116 human tumour cell lines. Tumour cells were treated with various concentrations (1–100 μg/mL) of EO for 72 h and then submitted to the MTT assay. As shown in Table 4, the EO showed notable growth inhibition effects on all human tumour cells in a dose-dependent manner (*p* < 0.05). The inhibition was remarkable with IC_50_ values of 5.51, 5.96 and 8.45 μg/mL on A375, MDA-MB 231 and HCT116, respectively. These IC_50_ values are commonly considered as promising for anticancer drug discovery and development. In fact, according to the standards of the National Cancer Institute (NCI), a plant extract may be considered as active and potential source of anticancer drugs if its IC_50_ is lower than 20 μg/mL (Cordell et al. 1993). The observed cytotoxicity was not specific toward a cancer cell line and it cannot be attributed to a compound or few compounds as shown in the composition of EO. In order to compare the obtained results with literature we considered the previously published data on some of the main constituents. (*E*)-nerolidol, the main compound present in *T. alternans* EO, has been shown to be cytotoxic on renal cell adenocarcinoma ACHN, the hormone-dependent prostate carcinoma LNCaP, human lung carcinoma A-549 and colon adenocarcinoma DLD-1 (Loizzo et al. 2007; Sylvestre et al. 2007). The IC_50_ values of this compound determined by us on tumour cell lines were 2.92, 4.13 and 5.76 μg/mL for A375, HCT116 and MDA-MB 231, respectively, but the differences in the various types of tumour cells and the possible variation in the assay protocol should be also considered. The antiproliferative activity of neral and geranial, the two isomers of citral, were tested in our previous study (Maggi et al. 2013), and IC_50_ values ranged from 5.73 to 11.3 μg/mL on MDA-MB 231 and A375, respectively. Germacrene D has been reported to inhibit the growth of MDA-MB-231, MCF7, Hs 578T, PC-3, and Hep-G2 cell lines (Kuźma et al. 2009). Neryl acetate and nerol were also assayed and showed lower cytotoxic activity against the tested tumour cell lines (Table 4). No data are available in the literature regarding cytotoxic activity of (*E*)-β-ocimene. The concentrations of (*E*)-nerolidol (15.8%), (*E*)-β-ocimene (7%), geranial (6.8%), germacrene D (6.7%), and neral (5.1%) cannot fully explain the observed cytotoxic activity. This means that other minor compounds contributed to the activity, taking into account that synergism between constituents may increase the total antiproliferative activity exhibited by the EO. *T. alternans* EO presented an excellent inhibitory activity against human tumour cell lines with IC_50_ values close to those of the cisplatin (*p* < 0.05). These results suggest further investigations on the possible mechanisms of action.

**Table 4.** *In vitro* cytotoxic activity of *T. alternans* EO^a^.

IC_50_ (μg/ml) ± S.D.^b^			
Compound	A375^c^	MDA-MB 231^d^	HCT116^e^
Essential oil	5.51 ± 0.34	5.9 6 ± 0.46	8.45 ± 0.61
(*E*)-Nerolidol	2.92 ± 0.09	5.76 ± 0.29	4.13 ± 0.18
	(13.13 ± 0.43)	(25.90 ± 1.31)	(18.57 ± 0.81)
Neryl acetate	28.05 ± 1.27	27.37 ± 1.87	45.61 ± 2.83
	(142.9 ± 6.47)	(139.5 ± 9.53)	(232.4 ± 14.4)
Nerol	75.83 ± 3.97	24.44 ± 1.66	33.49 ± 4.53
	(491.6 ± 25.7)	(158.4 ± 10.8)	(217.1 ± 29.4)
Positive control			
Cisplatin	0.43 ± 0.045	2.94 ± 0.23	2.42 ± 0.21
	(1.43 ± 0.15)	(9.80 ± 0.76)	(8.06 ± 0.70)

a Sample was obtained from population growing in Nová Sedlica.

b IC_50_= The concentration of compound that affords a 50% reduction in cell growth (after 72 h of incubation).

c Human malignant melanoma cell line.

d Human breast adenocarcinoma cell line.

e Human colon carcinoma cell line. Bracketed IC_50_ values are expressed in μM ± S.D.

## Conclusions

Phytochemical investigations on *T. alternans* allowed identification of the main phytoconstituents of the plants aerial parts, showing the presence of glycosylated flavonoids and rosmarinic acid, pentacyclic triterpenes and EO. Triterpenes and EO exhibited significant antiproliferative activity against a panel of tumour cell lines. In particular, the pentacyclic triterpenes showed improved activity compared to the reference compounds cisplatin and betulinic acid. (*E*)-nerolidol, the main volatile component of *T. alternans* EO, appeared to afford the major contribution to the cytotoxic activity of the EO. This work pointed out the great chemical diversity of *T. alternans* phytocomplex. In addition, the significant cytotoxic activity of some of the isolated triterpenes underlines the possible role of the plant as a potential source of bioactive compounds for therapeutical applications.

## Supplementary Material

Stefano_Dall_Acqua_et_al_supplemental_content.zip

## References

[CIT0001] Adams R. 2007 Identification of essential oil components by gas chromatography/mass spectrometry. 4th ed Carol Stream, IL, USA: Allured Publishing Corp.

[CIT0002] Agrawal PK, Bansal MC. 1989 Carbon-13 NMR of flavonoids. New York, USA: Elsevier; pp. 283–363.

[CIT0003] Ahiahonu PWK, Goodenowe DB. 2007 Triterpenoids from leaves of *Elaeophorbia drupifera*. Fitoterapia. 78:337–341. 17507177 10.1016/j.fitote.2007.02.002

[CIT0004] Arruda DC, Alexandri FLD, Katzin AM, Uliana SRB. 2005 Antileishmanial activity of the terpene nerolidol. Antimicrob Agents Chemother. 49:1679–1687. 15855481 10.1128/AAC.49.5.1679-1687.2005 PMC1087654

[CIT0005] Baser KHC, Demirci B, Kürçüoglu M, Tümen G. 1999 Essential oil of *Thymus zygioides* Griseb. var. *zygioides* from Turkey. J Essent Oil Res. 11:409–410.

[CIT0006] Chudzik M, Korzonek-Szlacheta I, Król W. 2015 Triterpenes as potentially cytotoxic compounds. Molecules. 20:1610–1625. 25608043 10.3390/molecules20011610 PMC6272502

[CIT0007] Claire EL, Schwaiger S, Banaigs B, Stuppner H, Gafner F. 2005 Distribution of a new rosmarinic acid derivative in *Eryngium alpinum* L. and other Apiaceae. J Agric Food Chem 53:4367–4372. 15913297 10.1021/jf050024v

[CIT0008] Cordell GA, Kinghorn D, Pezzuto JM. 1993 Separation, structure elucidation, and bioassay of cytotoxic natural products In Colegate SM, Molyneux RJ, editors. Bioactive natural products. Boca raton: CRC Press; p. 195–216.

[CIT0009] de Assis Lage TC, Montanari RM, Fernandes SA, de Oliveira Monteiro CM, de Oliveira Souza Senra T, Zeringota V, da Silva Matos R, Daemon E. 2015 Chemical composition and acaricidal activity of the essential oil of *Baccharidis dracunculifolia* De Candole (1836) and its constituents nerolidol and limonene on larvae and engorged females of *Rhipicephalus microplus* (Acari: Ixodidae). Exp Parasitol. 148:24–29. 25448290 10.1016/j.exppara.2014.10.011

[CIT0010] European Pharmacopoeia 2008. 6.0 Edition, Council of Europe, Strassbourg, 2008, vol. I–II.

[CIT0011] FFNSC 2 2012. Flavors and fragrances of natural and synthetic compounds. Mass spectral database. Shimadzu Corps, Kyoto.

[CIT0012] Fonsêca DV, Salgado PRR, de Carvalho FL, Salvadori MGSS, Penha ARS, Leite FC, Borges CJS, Piuvezam MR, de Morais Pordeus LC, Sousa DP, et al 2016 Nerolidol exhibits antinoceptive and anti-inflammatory activity: involvement of the GABAergic system and proinflammatory cytokines. Fund Clin Pharmacol. 30:14–22. 10.1111/fcp.12166 26791997

[CIT0013] Fulda S, Kroemer G. 2009 Targeting mitochondrial apoptosis by betulinic acid in human cancers. Drug Discov Today 14:885–890. 19520182 10.1016/j.drudis.2009.05.015

[CIT0014] Gosh P, Mandal A, Rasul MG. 2013 A new bioactive ursane-type triterpenoid from *Croton bonplandianum* Bail. J Chem Sci 125:359–364.

[CIT0015] Green SA, Chen X, Nieuwenhuizen NJ, Matich AJ, Wang MY, Bunn BJ, Yauk Y, Atkinson RG. 2011 Identification, functional characterization, and regulation of the enzyme responsible for floral (*E*)-nerolidol biosynthesis in kiwifruit (*Actinidia chinensis*). J Exp Bot. 63:1951–1968. 22162874 10.1093/jxb/err393 PMC3295389

[CIT0016] Jamali CA, El Bouzidi L, Bekkouche K, Lahcen H, Markouk M, Wohlmuth H, Leach D, Abbad A. 2012 Chemical composition and antioxidant and anticandidal activities of essential oils from different wild Moroccan *Thymus* species. Chem Biodivers. 9:1188–1197. 22700236 10.1002/cbdv.201200041

[CIT0017] Jamzad M, Rustaiyan A, Jamzad Z, Masoudi S. 2011 Essential oil composition of *Salvia indica* L., *Thymus caucasicus* Wind. Ex Ronniger subsp. *grossheimii* (Ronniger) Jalas and *Ballota nigra* L. three Labiatae species from Iran. J Essent Oil-Bear Plants. 14:76–83.

[CIT0018] Keeler RF, Tu AT. 1991 Toxicology of plant and fungal compounds. Handbook of natural toxins. Marcel Dekker. 6: 665.

[CIT0019] Klokov MV. 1960 Rid 747. Čebrec – Thymus L In: Klokov M.V. Editor. Flora URSR IX. Kiev: Naukovaja Dumka; p. 294–348.

[CIT0020] Kuźma Ł, Kalemba D, Różalski M, Różalska B, Więckowska-Szakiel M, Krajewska U, Wysokińska H. 2009 Chemical composition and biological activities of essential oil from *Salvia sclarea* plants regenerated *in vitro*. Molecules. 14:1438–1447. 19384275 10.3390/molecules14041438 PMC6254371

[CIT0021] Lee K, Lee JH, Kim SI, Cho MH, Lee J. 2014 Anti-biofilm, anti-hemolysis, and anti-virulence activities of black pepper, cananga, myrrh oils, and nerolidol against *Staphylococcus aureus*. Appl Microbiol Biotechnol. 98:9447–9457. 25027570 10.1007/s00253-014-5903-4

[CIT0022] Loizzo MR, Tundis R, Statti GA, Menichini F. 2007 Jacaranone: a cytotoxic constituent from *Senecio ambiguus* subsp. *ambiguus* (Biv.) DC. against renal adenocarcinoma ACHN and prostate carcinoma LNCaP cells. Arch Pharm Res. 30:701–707. 17679547 10.1007/BF02977631

[CIT0023] Maggi F, Fortuné Randriana R, Rasoanaivo P, Nicoletti M, Quassinti L, Bramucci M, Lupidi G, Petrelli D, Vitali LA, Papa F, et al 2013 Chemical composition and *in vitro* biological activities of the essential oil of *Vepris macrophylla* (Baker) I. Verd. endemic to Madagascar. Chem Biodivers. 10:356–366. 23495153 10.1002/cbdv.201200253

[CIT0024] Maggi F, Caprioli G, Papa F, Sagratini G, Vittori S, Kolarcik V, Mártonfi P. 2014 Intra-population chemical polymorphism in *Thymus pannonicus* all growing in Slovakia. Nat Prod Res. 28:1567–1573. 24934518 10.1080/14786419.2014.926355

[CIT0025] Mártonfi P. 1996 *Thymus alternans* Klokov – a new species of Slovak flora. Biologia (Bratislava). 51:27–29.

[CIT0026] Mártonfi P, Mártonfiová L. 1996 *Thymus* chromosome numbers from Carpathians and Pannonia. Thaiszia – J. Bot 6:25–38.

[CIT0027] Mockute D, Bernotiene G. 2005 Chemical composition of the essential oils and the odor of *Thymus pulegioides* L. growing wild in Vilnius. J Essent Oil Res. 17:415–418.

[CIT0028] Morales R. 2002 The history, botany and taxonomy of the genus *Thymus* In: Stahl-Biskup E, Saez F, Editors. Thyme: the genus thymus. London: Taylor & Francis; p. 1–43.

[CIT0029] Nabavi SM, Marchese A, Izadi M, Curti V, Daglia M, Nabavi SF. 2015 Plants belonging to the genus *Thymus* as antibacterial agents: from farm to pharmacy. Food Chem. 173:339–347. 25466031 10.1016/j.foodchem.2014.10.042

[CIT0030] Načhyčhko V. 2014 The genus *Thymus* l. (Labiatae Juss.) in the Ukrainian Carpathians flora: systematics and taxonomic problems. Visn Lviv Univ Ser Biol. 64:159–169.

[CIT0031] NIST 08 2008. Mass spectral library (NIST/EPA/NIH). National Institute of Standards and Technology, Gaithersburg, USA.

[CIT0032] Nogueira Neto JD, De Almeida AAC, Da Silva Oliveira J, Dos Santos PS, De Sousa DP, De Freitas RM. 2013 Antioxidant effects of nerolidol in mice hippocampus after open field test. Neurochem Res. 38:1861–1870. 23765368 10.1007/s11064-013-1092-2

[CIT0033] Shan H, Wilson WK, Castillo DA, Matsuda SP. 2015 Are isoursenol and γ-amyrin rare triterpenes in nature or simply overlooked by usual analytical methods? Org Lett 21. 17:3986–3989. 10.1021/acs.orglett.5b01851 26235440

[CIT0034] Sylvestre M, Pichette A, Lavoie S, Longtin A, Legault J. 2007 Composition and cytotoxic activity of the leaf essential oil of *Comptonia peregrina* (L.) Coulter. Phytother Res. 21:536–540. 17326040 10.1002/ptr.2095

[CIT0035] Tümen G, Kirimer N, Kurkcuoglu M, Baser KHC. 1997 Composition of the essential oils of *Thymus atticus* and *Thymus roegneri* from Turkey. J Essent Oil Res. 9:473–474.

[CIT0036] Tzakou O, Constantinidis T. 2005 Chemotaxonomic significance of volatile compounds in *Thymus samius* and its related species *Thymus atticus* and *Thymus parnassicus*. Biochem Syst Ecol. 33:1131–1140.

[CIT0037] Vidic D, Ćavar S, Šolić ME, Maksimović M. 2010 Volatile constituents of two rare subspecies of *Thymus praecox*. Nat Prod Commun. 5:1123–1126. 20734955

[CIT0038] Wattenberg LW. 1991 Inhibition of azoxymethane-induced neoplasia of the large bowel by 3-hydroxy-3,7,11-trimethyl-l,6,10-dodecatriene (nerolidol). Carcinogenesis. 12:151–152. 1988176 10.1093/carcin/12.1.151

[CIT0039] Zhang Y, Nakamura S, Wang T, Matsuda H, Yoshikawa M. 2008 The absolute stereostructures of three rare D:B-friedobaccharane skeleton triterpenes from the leaves of *Salacia chinensis*. Tetrahedron. 64:7347–7352.

