# Supplementary material for: Phytochemical investigations and antiproliferative secondary metabolites from Thymus alternans growing in Slovakia
Source: Pharm Biol. 2017 Feb 21;55(1):1162–70. doi: 10.1080/13880209.2017.1291689 (PMC6130594; doi:10.1080/13880209.2017.1291689)
Supplement: Stefano_Dall_Acqua_et_al_supplemental_content.zip [file IPHB_A_1291689_SM6858.zip › Supplementary information.docx]

**Phytochemical investigations and antiproliferative secondary metabolites from *Thymus alternans* growing in Slovakia**

Stefano Dall’Acqua^a*^, Gregorio Peron^a^, Sara Ferrari^a^, Valentina Gandin^a^, Massimo Bramucci^b^, Luana Quassinti^b^, Pavol Mártonfi^c^, Filippo Maggi^b^

^a^Department of Pharmaceutical and Pharmacological Sciences, University of Padova, Italy

^b^School of Pharmacy, University of Camerino, Italy

^c^Institute of Biology and Ecology, Department of Botany, P. J. Šafárik University, Košice, Slovakia

**Supplementary information**

NMR assignments for isolated compounds

NMR assignments for compounds P1-P4

|  | ***P1*** |  | ***P2*** |  | ***P3*** |  | ***P4*** |  |
| --- | --- | --- | --- | --- | --- | --- | --- | --- |
| ***Position*** | ***δH*** | ***δC*** | ***δH*** | ***δC*** | ***δH*** | ***δC*** | ***δH*** | ***δC*** |
| *1* | - | - | - | - | - | - | - | - |
| *2* | - | 162.0 | - | 163.0 | - | 163.0 | - | 165,0 |
| *3* | 6.70 | 104.3 | 6.71 | 104.8 | 6.60 | 104.4 | 6,65 | 114,7 |
| *4* | - | 180.6 | - | 181.4 | - | 182.0 | - | 180,6 |
| *5* | - | 162.2 | - | 161.0 | - | 157.5 | - | 163,2 |
| *6* | 6.15 | 97.7 | 6.25 | 98.1 | 6.30 | 102.0 | 6,82 | 105,7 |
| *7* | - | 164.9 | - | 165.0 | - | 163.1 | - | 161,5 |
| *8* | 6.48 | 96.0 | 6,77 | 92.4 | 6,82 | 93.7 | 6,51 | 104,7 |
| *9* | - | 158.5 | - | 158.1 | - | 158.4 | - | 158,6 |
| *10* | - | 103.0 | - | 103.1 | - | 103.1 | - | 105,8 |
| *1’* | - | 123.0 | - | 123.2 | - | 122.5 | - | 121,8 |
| *2’* | 6.76 | 127.0 | 6.72 | 112.0 | 6.75 | 112.5 | 7,40 | 115,9 |
| *3’* | - | 144.8 | - | 148.8 | - | 147.8 | 7,08 | 111,0 |
| *4’* | - | 145 | - | 147.5 | - | 148.5 | - | 161,0 |
| *5’* | 6.85 | 117.6 | 7.15 | 117.0 | 7.36 | 115.6 | 6,92 | 115,9 |
| *6’* | 7.37 | 121.0 | 6.95 | 121.0 | 7.35 | 120.5 | 7,90 | 127,4 |
| *OCH3* | - | - | 3.95 | 54.5 | 3.92 | 55.2 | - | - |
| *1-gluc* | 4.88 | 102.4 | 4,85 | 103,4 | 4,82 | 103.0 | 5,10 | 100,4 |
| *2-gluc* | 3.55 | 76.6 | 3,5 | 76,5 | 3,58 | 76,5 | 3,54 | 76,1 |
| *3-gluc* | 3.48 | 70.6 | 3,48 | 76,5 | 3,45 | 74,5 | 3,60 | 70,4 |
| *4-gluc* | 3.42 | 76.2 | 3,50 | 76,2 | 3,46 | 75,2 | 3,58 | 76,7 |
| *5-gluc* | 3.62 | 73.2 | 3,62 | 73,1 | 3,59 | 70,1 | 3,62 | 73,2 |
| *6-gluc* | 3.79-3.25 | 60.7 | 3,95-3.49 | 61.0 | 3,85-3.60 | 60,2 | 3,87 | 60,5 |

NMR assignments for compounds P5-P7

|  | ***P5*** |  | ***P6*** |  | ***P7*** |  |
| --- | --- | --- | --- | --- | --- | --- |
| ***Position*** | ***δH*** | ***δC*** | ***δH*** | ***δC*** | ***δH*** | ***δC*** |
| *1* | - | 109,4 | - | 127,5 | - |  |
| *2* | 7,05 | 113,6 | 7,08 | 113,6 | - | 114,06 |
| *3* | - | 148,1 | - | 148,2 | - | 149,8 |
| *4* | - | 148,1 | 7,05 d | - | - | 150 |
| *5* | 6,79 | 115,2 | 6,80 | 115,2 | 7,07 | 117,6 |
| *6* | 6,96 | 121,9 | 6,96 | 121,9 | 6,68 | 115,8 |
| *7* | 7,51 | 146,2 | 5,51 | 146,2 | 7,89 | 144,5 |
| *8* | 6,26 | 113,4 | 6,26 d | 114,1 | 6 | 115,6 |
| *9* | - | 167,8 | - | 167,8 | - | 171,3 |
| *1’* | - | 109,4 | - | 127,9 |  |  |
| *2’* | 6,70 | 116,3 | 6,65 d | 116,3 |  |  |
| *3’* | - | 144,5 | - | 143,5 |  |  |
| *4’* | - | 144,5 | - | 149,2 |  |  |
| *5’* | 6,70 | 115,0 | 6,76 | 115,2 |  |  |
| *6’* | 6,61 | 120,6 | 6,80 | 120,4 |  |  |
| *7’* | 2,99 | 36,4 | 3,03 | 36,4 |  |  |
| *8’* | 5,20 | 73,4 | 5,23 m | 74,0 |  |  |
| *9’* | - | 172,4 | - | 171,4 |  |  |
| *1-gluc* |  |  | 4,30 | 101,96 | 4,37 | 102,5 |
| *2-gluc* |  |  | 3,22 | 73,2 | 3,40 | 75,3 |
| *3-gluc* |  |  | 3,48 | 73,53 | 3,45 | 73,1 |
| *4-gluc* |  |  | 3,36 | 76,6 | 3,46 | 76,3 |
| *5-gluc* |  |  | 3,32 | 69,8 | 3,41 | 72,0 |
| *6-gluc* |  |  | 3,80 | 61,4 | 3,91 | 61,3 |

NMR assignments for compounds T1 and T5

| ***Posizione*** | ***δH*** | ***δC*** | ***δH*** | ***δC*** |
| --- | --- | --- | --- | --- |
| *1* | 0,99 | 40.5 | 1.00 | 38.5 |
| *2* | 1.80 | 29.7 | 1.57 | 27,4 |
| *3* | 3.46 | 71.8 | 3.45 | 77.4 |
| *4* | - | 33.9 | - | 34.2 |
| *5* | 0.88 | 54.7 | 0.88 | 55.3 |
| *6* | 1.40 | 18,4 | 1.56 | 19.2 |
| *7* | 1.50-1.80 | 31.9 | 1.34 | 41.3 |
| *8* | - | 39.7 | - | 39.2 |
| *9* | 1.54 | 50.1 | 1.44 | 49.4 |
| *10* | - | 37.2 | - | 37.6 |
| *11* | 1,89 | 23,7 | 1.65 | 17.5 |
| *12* | 5,27 | 122.0 | 1.55 | 33.9 |
| *13* | - | 143,6 | - | 37.6 |
| *14* | - | 42.4 | - | 158.0 |
| *15* | 5.11 | 128.9 | 5.40 | 116.3 |
| *16* | 5.11 | 134.0 | 1.92-1.60 | 37.8 |
| *17* | - | 45.2 | - | 35.8 |
| *18* | 2.30 | 54.2 | 0.95 | 48.8 |
| *19* | 2.21 | 56.0 | 1.30 | 36.7 |
| *20* | 1.43 | 56.8 | - | 28.9 |
| *21* | 1.50 | 31.6 | 1.30 | 33.1 |
| *22* | 2.00 | 37.2 | 1.40 | 35.2 |
| *23* | 0.68 | 21.1 | 0.85 | 33.4 |
| *24* | 0.69 | 29.1 | 0.83 | 21.6 |
| *25* | 0.76 | 28.2 | 0.92 | 15.4 |
| *26* | 0.84 | 19.8 | 1.09 | 26.0 |
| *27* | 1.01 | 26.0 | 0.93 | 21.4 |
| *28* | 1.25 | 19.4 | 0.82 | 29.8 |
| *29* | 0.79 | 19.0 | 0.95 | 33.4 |
| *30* | 0.92 | 23.0 | 0.90 | 29.9 |
